# Leptin Promotes HTR-8/SVneo Cell Invasion via the Crosstalk between MTA1/WNT and PI3K/AKT Pathways

**DOI:** 10.1155/2022/7052176

**Published:** 2022-11-22

**Authors:** Minghua Fan, Lihua Dong, Yanping Meng, Yao Wang, Junhui Zhen, Jianqing Qiu

**Affiliations:** ^1^Department of Obstetrics and Gynecology, The Second Hospital, Cheeloo College of Medicine, Shandong University, Jinan, Shandong 250033, China; ^2^Department of Obstetrics and Gynecology, Qilu Hospital, Cheeloo College of Medicine, Shandong University, Jinan, Shandong 250012, China; ^3^Department of Pathology, School of Medicine, Shandong University, Jinan, 250021 Shandong, China

## Abstract

The process of placental invasion is essential for a successful pregnancy. Leptin is involved in trophoblast invasiveness, and its dysregulation is connected with a series of diseases, including preeclampsia. However, the knowledge of the precise mechanisms in leptin-induced trophoblast invasiveness is still limited. According to the present research, transwell assay suggested that leptin is a dose- and time-dependent regulator in inducing HTR-8/SVneo cell invasion. Western blot analysis and immunofluorescence staining revealed that leptin-induced MMP9 expression is essential in the invasion process of HTR-8/SVneo cells. Mechanistically, we demonstrated that leptin activated *β*-catenin via the crosstalk between the MTA1/WNT and PI3K/AKT pathways. Besides, we showed that downregulating the key molecules in the signaling pathways by siRNA can inhibit leptin-induced MMP9 expression and further suppress invasion of HTR-8/SVneo cells. In conclusion, our study revealed a new regulatory mechanism of leptin-induced HTR-8/SVneo cell invasiveness and will provide novel insights into the causes and potential therapeutic targets for diseases related to dysregulation of trophoblast invasion in the future.

## 1. Introduction

At the early stage of pregnancy, the invasion of trophoblast cells into the uterus is essential for implantation and subsequent placental development [1]. The invasion of extravillous trophoblasts includes recognition, adhesion, matrix degradation, penetration of basal membrane, and invasion in uterine wall, which is regulated by intricate and comprehensive factors [1]. If the invasion is insufficient, a series of pregnancy complications may occur, such as growth retardation, miscarriage, or preeclampsia [2–4]. Besides, it can cause placenta accreta while the invasion into the myometrium is excessive [5]. Therefore, the invasion process must be precisely regulated. However, previous studies did not well identify the exact mechanisms of placenta invasion. As a result, the intensive study targeting to the molecular mechanisms of placental invasion is necessary to the diagnosis and therapy in clinic.

Discovered at the end of 1994, Leptin is a 16 kDa polypeptide hormone containing 167 amino acids, which is produced by the obese gene [6]. Studies have reported that leptin not only regulates appetite and energy expenditure at the hypothalamic level but also plays an important role in inflammation, reproduction, and angiogenesis through transmembrane leptin receptor (leptin-R) [7–10]. During pregnancy, leptin is respectively synthesized by adipose tissue and the placenta, leading to more elevated levels of circulating leptin than in prepregnancy state [11–13]. Hence, the dysregulation of leptin is liable to inducing the reproductive and gestating disorders. In recent years, accumulating evidence implied the role of leptin in the regulation of trophoblast invasiveness [14–16]. However, how the leptin affects cytotrophoblast invasion is still not clear.

As a kind of calcium-dependent, zinc-containing endopeptidases, matrix metalloproteinases (MMPs) are essential to the degradation of the extracellular matrix (ECM) and successful implantation [17]. Studies have shown that MMPs are important to placental development, inflammation, angiogenesis, tumor invasion, and metastasis in the physiological and pathophysiological processes [18–20]. In addition, MMP9 is crucial for trophoblast invasion [21, 22], and aberrant expression of MMP9 in extravillous trophoblasts is linked to preeclampsia [23]. Furthermore, leptin was also reported to participate in the invasive processes by modulating the expression of MMPs [24]. Thus, studying the mechanism regulating the leptin-induced MMP9 expression will provide novel insights into the underlying matrix degradation and extravillous trophoblast invasion in the future.

Acting as a nuclear transcriptional regulator, *β*-catenin regulates proliferation, migration, and differentiation. And there have been studies demonstrated that *β*-catenin can promote the trophoblast hyperplasia and invasion [25, 26]. In humans, there are three WNT signaling pathways, including canonical WNT/*β*-catenin pathway, noncanonical WNT/Ca^2+^ pathway, and noncanonical planar cell polarity pathway. Among them, WNT/*β*-catenin is the most widely studied, and its aberrant activation has been reported in a variety of diseases, including invasion [27–30]. MTA1, the first discovered number of metastasis-associated gene (MTA) family, acts as a cancer progression-related genes in the invasion and metastasis of breast, ovarian, gastrointestinal, and colorectal cancer [31, 32]. However, whether MTA1 could mediate WNT/*β*-catenin signaling in trophoblast cells remains largely unknown.

Phosphatidylinositol 3-kinase (PI3K) is a kind of enzyme that phosphorylates the 3′-OH of the inositol ring of phosphatidylinositol [33]. When it is activated, it can induce the production of phosphatidylinositol 3,4,5-triphosphate (PIP3), leading to activation of the serine/threonine kinases AKT. PI3K/AKT pathway is reported as the regulator of numerous cellular functions including proliferation, invasion, metabolism, and angiogenesis [33]. However, whether PI3K/AKT pathways play a role in trophoblast cell invasion and whether there exists a crosstalk between WNT/*β*-catenin and PI3K/AKT pathways in trophoblast cell invasion need to be elucidated.

Taken together, our study manages to investigate the effect of leptin on HTR-8/SVneo cell invasion and the potential underlying mechanisms.

## 2. Materials and methods

### 2.1. Reagent and Cell Culture

Recombinant human leptin (Solarbio, China) was dissolved in ddH_2_O to 100 *μ*g/ml. HTR-8/SVneo cells were cultured in RPMI 1640 Medium (Gibco, USA) supplemented with 10% fetal bovine serum (FBS) (Gibco, USA) and 1% penicillin-streptomycin in a humidified atmosphere containing 5% CO_2_ at 37°C and then treated with or without leptin.

### 2.2. Cell Transfection

The MTA1 siRNA, AKT1 siRNA, WNT1 siRNA, and *β*-catenin siRNA were synthesized by Genomeditech (Shanghai, China). And transfection was performed according to the manufacturer's protocols. The target sequences were designed as the following: MTA1 siRNA: 5′-GAACAUCUACGACAUCUCC-3′; AKT1 siRNA: 5′-GACGGGCACAUUAAGAUCA-3′; WNT1 siRNA: 5′-GGUUCCAUCGAAUCCU-

GCA-3′; *β*-catenin: 5′-CCUUCACUAUGGACUACCA-3′; MMP9: 5′-CACGCACGACGUCUUCCAGUA-3′; negative control siRNA: 5′-UUCUCCGAACGUGUCACGU-3′. The transfection was performed by using Lipofectamine 3000 reagent (Thermo Fisher Scientific, USA).

### 2.3. Transwell Assay

Transwell Matrigel invasion assay was used to measure the capacity of HTR-8/SVneo cell invasion. Briefly, dilute Matrigel (1 : 4) (BD Biosciences, USA) in serum-free RPMI 1640 medium and put 50 *μ*l of the diluted Matrigel into the upper chamber of 24-well transwell inserts (8 *μ*m pores; BD Biosciences) then incubate the transwell at 37°C 3-4 h for gelling. Cells suspended in 100 *μ*l serum-free RPMI 1640 medium were added to the upper chamber. 600 *μ*l RPMI 1640 medium containing 10% FBS was added into the lower chamber. After being treated with different interventions for intended time at 37°C, cells were fixed with 4% paraformaldehyde and penetrated with 0.3% Triton X-100. After removing noninvading cells, dying with hematoxylin, the cells were counted with a light microscope (200x, magnification).

### 2.4. Wound-Healing Assay

HTR-8/SVneo cells were seeded into 6-well plates, and when confluence reached to 90%, cells were scratched vertically with the same width. Then, wash away the scratched cells before putting the rest into the incubator for further culture. Photographs of five random fields in each chamber were obtained at 0 h and 24 h after scratch. And the percentage of wound closure was analyzed by calculating (*A* − *B*)/*A* × 100%. *A* and *B* represent the scratch width after cell migration at 0 and 24 h, respectively.

### 2.5. RNA Extraction and Quantitative Real-Time PCR (qRT-PCR) Analysis

Total RNA was extracted from HTR-8/SVneo cells using TRIzol reagent (Takara, Japan), and cDNA was synthesized using PrimeScript RT Reagent Kit (Takara, Japan). According to the manufacturer's protocol, the qPCR reaction was performed on LightCycler® 480 Real-Time PCR System (Roche, USA). The sequences of the primers used in qRT-PCR are listed in Table 1. *β*-Actin was used as the internal control, and the relative expression of target genes were calculated using the 2^−∆∆CT^ method.

### 2.6. Western Blot Analysis

HTR-8/SVneo cells grown to confluency were washed with ice-cold PBS three times and lysed in RIPA buffer (Beyotime Biotechnology, China) containing proteinase inhibitors and phosphatase inhibitors (Solarbio, China). After being denatured, equal protein (about 20 *μ*g) was separated using SDS-PAGE and transferred to PVDF membranes (Millipore, USA). The membranes were then blocked with 5% milk or BSA for 2 h at room temperature and incubated with primary antibodies overnight at 4°C. Following incubation with the secondary antibody at room temperature for 1 h, the level of the proteins was quantified using ECL reagent (MilliporeSigma, USA) and imaged by the Amersham Imager 600 (GE, USA). *β*-Actin was used as the control of total proteins. Nuclear protein was extracted with a Nuclear and Cytoplasmic Extraction Kit (CW0199, CoWin BioSciences, China), and histone 3 was used as the control of nuclear proteins. The following primary antibodies were used: MMP9 (ab76003), MTA1 (ab71153), WNT1 (ab15251), AKT (ab179463), GSK3*β* (ab32391), *β*-catenin (ab32572), histone 3 (ab1791), *β*-actin (ab6276) (Abcam, USA); p-AKT (Ser473) (#4060), p-GSK3*β* (Ser9) (#9322) (Cell Signaling Technology, USA).

### 2.7. Immunofluorescence Staining

HTR-8/SVneo cells were seeded in the 6-well chamber slides followed by different interventions. The treated cells were fixed with 4% paraformaldehyde, penetrated with 0.1% Triton X-100, blocked with 5% BSA for 30 min at room temperature, and then incubated with primary antibody at 4°C overnight. After washing with PBS for three times, cells were dyed with DyLight 594-conjugated IgG (ab150080, Abcam, USA) for 1 h at 37°C under dark conditions. Next, cells were washed with PBS in triplicate, followed by incubation with DAPI for 10 min. Finally, cells were observed under a fluorescence microscope (Olympus, Japan).

### 2.8. Statistical Analysis

Data analysis was performed with GraphPad Prism 8 and Adobe Photoshop. The experimental results were presented as mean ± SD. Differences between the two groups were analyzed by Student's *t*-test while differences among multiple groups were analyzed by one-way ANOVA. *P* < 0.05 was considered a statistically significant difference. All experiments were conducted at least in triplicate.

## 3. Results

### 3.1. Leptin Exposure Induces HTR-8/SVneo Cell Invasion

**Figure 1:**
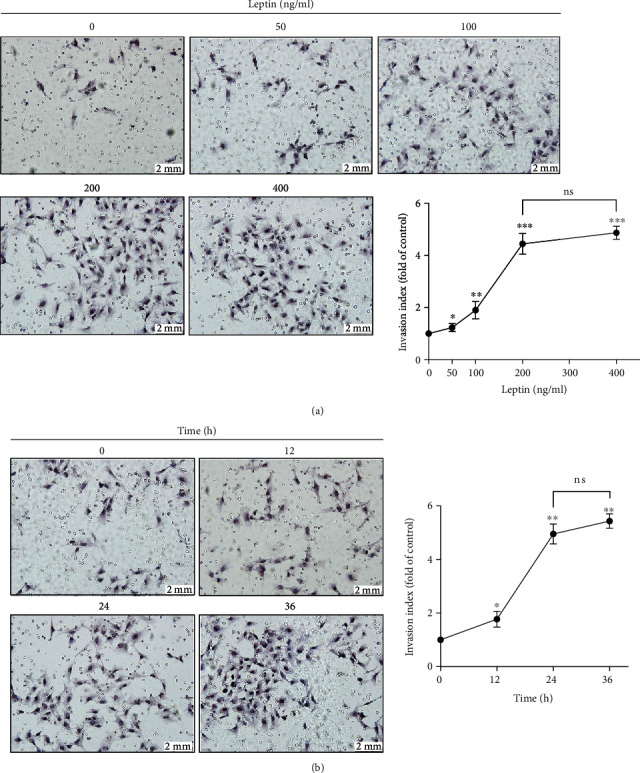
Leptin exposure induces HTR-8/SVneo cell invasion. (a) HTR-8/SVneo cells were treated with exogenous leptin (0, 50, 100, 200, and 400 ng/ml) for 24 h, and invasive potential was measured by transwell assay. Scale bar = 2 mm. ^∗^*P* < 0.05 vs. leptin (0 ng/ml), ^∗∗^*P* < 0.05 vs. leptin (50 ng/ml), and ^∗∗∗^*P* < 0.01 vs. leptin (100 ng/ml); ns: no significance. (b) HTR-8/SVneo cells were treated for 0 h, 12 h, 24 h, and 36 h with 200 ng/ml leptin, and transwell assay was conducted to analyze their invasive potential. Scale bar = 2 mm. ^∗^*P* < 0.05 vs. leptin (0 h) and ^∗∗^*P* < 0.01 vs. leptin (12 h); ns: no significance. Data are shown as mean ± SD. All experiments were performed in triplicate.

Previous studies have shown that leptin is involved in the cytotrophoblast invasion. To confirm whether leptin stimulation induced the invasion of HTR-8/SVneo cells, we divided the cells into two groups. One was treated with exogenous leptin (0, 50, 100, 200, and 400 ng/ml) for 24 h; the other was treated for 0 h, 12 h, 24 h, and 36 h with 200 ng/ml leptin. To detect the invasiveness of HTR-8/SVneo cells, both of the groups were performed under a transwell assay, and the results revealed that leptin can increase the HTR-8/SVneo cell invasion in a dose-dependent (Figure 1(a)) and time-dependent (Figure 1(b)) manner.

### 3.2. Leptin Induces HTR-8/SVneo Cell Invasion by Promoting MMP9 Expression

**Figure 2:**
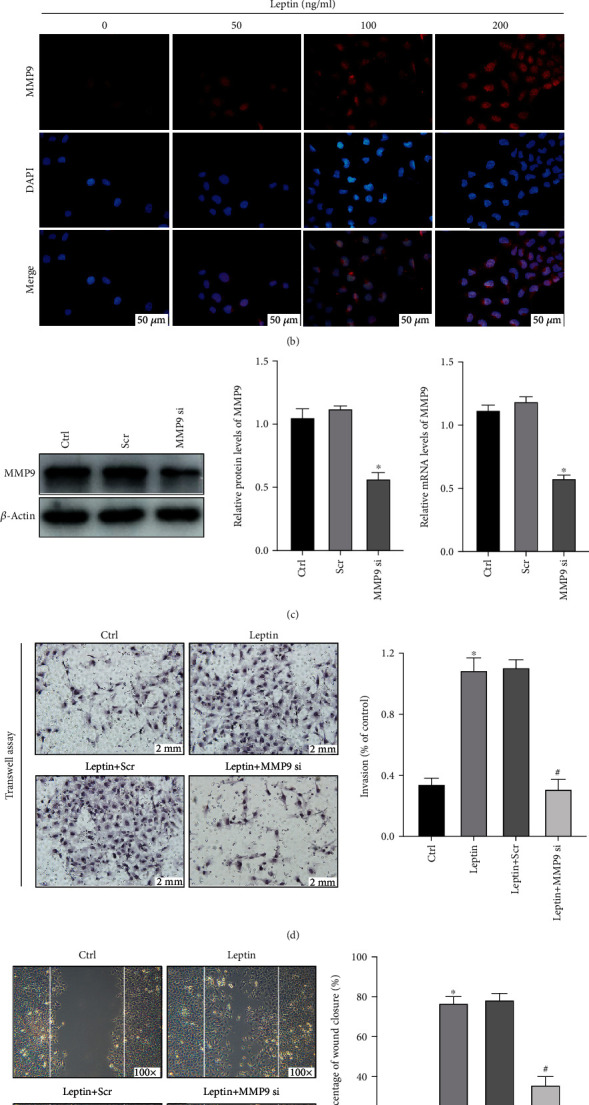
Leptin induces HTR-8/SVneo cell invasion by promoting MMP9 expression. (a) HTR-8/SVneo cells were treated with exogenous leptin (0, 50, 100, and 200 ng/ml) for 24 h, and MMP9 protein levels were measured by Western blot analysis. Data are shown as mean ± SD; ^∗^*P* < 0.01 vs. leptin (0 ng/ml), ^∗∗^*P* < 0.01 vs. leptin (50 ng/ml), and ^∗∗∗^*P* < 0.01 vs. leptin (100 ng/ml). (b) Representative immunofluorescence images of MMP9 in HTR-8/SVneo cells treated with exogenous leptin (0, 50, 100, and 200 ng/ml) for 24 h. Scale bar = 50 *μ*m. (c) The knockdown efficiency of MMP9 was analyzed by Western blot analysis and qRT-PCR. ^∗^*P* < 0.05 vs. control. (d) The results of transwell assay in HTR-8/SVneo cells treated with MMP9 siRNA or scramble siRNA (Scr) in the presence or absence of 200 ng/ml leptin for 24 h. Scale bar = 2 mm. Data are shown as mean ± SD; ^∗^*P* < 0.01 vs. control, ^#^*P* < 0.01 vs. leptin. (e) Results from the wound-healing assay are expressed as the percentage of wound closure. Magnification, 100x. Data are shown as mean ± SD; ^∗^*P* < 0.01 vs. control, ^#^*P* < 0.01 vs. leptin. All experiments were performed in triplicate.

Considering that MMP9 is crucial for trophoblast invasion, we explored the function of MMP9 in the process of leptin-induced invasion in HTR-8/SVneo cells. As shown in Figures 2(a) and 2(b), HTR-8/SVneo cells were incubated with (0, 50, 100, and 200 ng/ml) leptin for 24 h, and Western blot analysis and immunofluorescence staining demonstrated that leptin stimulation promoted MMP9 expression in HTR-8/SVneo cells. Meanwhile, with the increase of leptin concentration, the expression level of MMP9 is also enhanced. Subsequently, we silenced MMP9 with siRNA and confirmed the knockdown efficiencies with Western blot analysis and qRT-PCR (Figure 2(c)). By performing transwell assay and wound-healing assay, we observed that knockdown of MMP9 significantly alleviated leptin-induced invasion (Figures 2(d) and 2(e)), indicating that leptin induces HTR-8/SVneo cell invasion by promoting MMP9 expression.

### 3.3. Leptin Induced *β*-Catenin Activation in HTR-8/SVneo Cells

**Figure 3:**
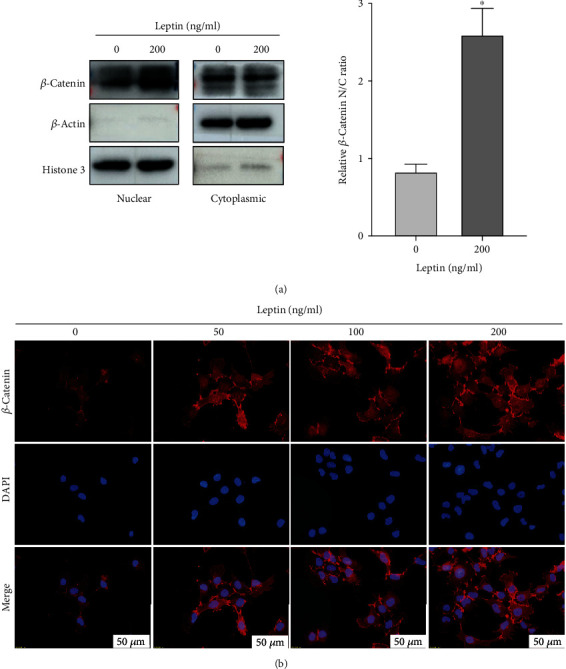
Leptin induced *β*-catenin activation in HTR-8/SVneo cells. (a) HTR-8/SVneo cells were treated with exogenous leptin (200 ng/ml) for 24 h, and the expression of *β*-catenin from nuclear and cytoplasmic fractions was detected by Western blot. Histone 3 was used as a nuclear marker and *β*-actin as a cytoplasmic marker. The relative *β*-catenin nuclear-to-cytoplasmic (N/C) ratio was calculated with nuclear *β*-catenin (normalized to histone 3) to cytoplasmic *β*-catenin (normalized to *β*-actin). Data are shown as mean ± SD; ^∗^*P* < 0.01 vs. leptin (0 ng/ml). (b) Representative immunofluorescence images of *β*-catenin in HTR-8/SVneo cells treated with exogenous leptin (0, 50, 100, and 200 ng/ml) for 24 h. Scale bar = 50*μ*m. All experiments were performed in triplicate.

To clarify the role of *β*-catenin in leptin-induced HTR-8/SVneo cell invasion, we detected the level of *β*-catenin with the stimulation of leptin. Firstly, treating HTR-8/SVneo cells with leptin (200 ng/ml) for 24 h, and the following Western blot showed that leptin increased the protein levels of nuclear *β*-catenin (Figure 3(a)). Besides, we administrated leptin (0, 50, 100, and 200 ng/ml) in HTR-8/SVneo cells for 24 h, and immunofluorescence staining indicated that leptin promoted the nuclear translocation of *β*-catenin in a dose-dependent manner, manifesting consistent result with Western blot analysis (Figure 3(b)). Collectively, these data suggested that leptin induced *β*-catenin activation.

### 3.4. Leptin Mediates *β*-Catenin Activation through the Crosstalk between MTA1/WNT and PI3K/AKT Pathways in HTR-8/SVneo Cells

**Figure 4:**
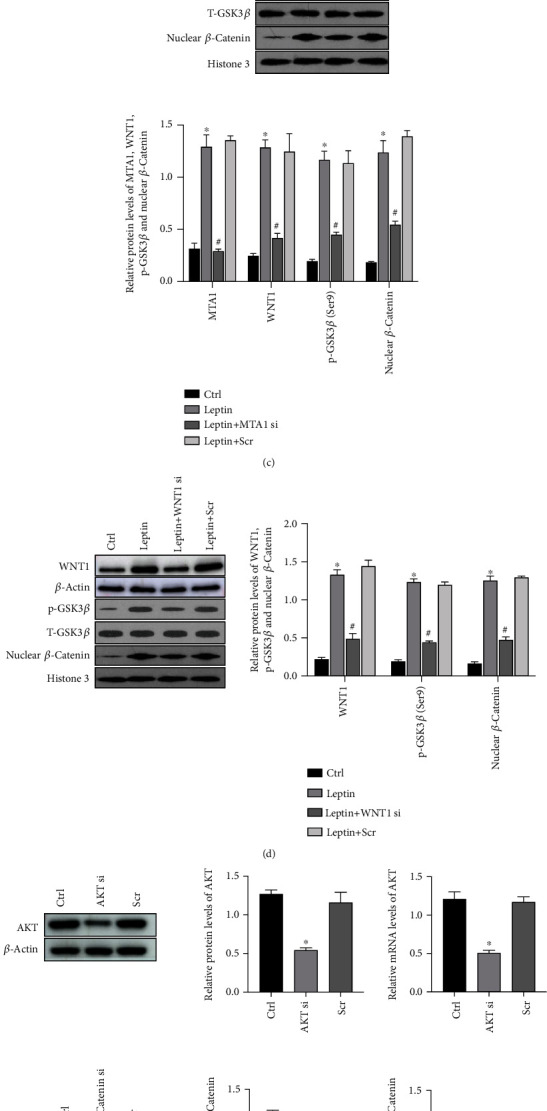
Leptin mediates *β*-catenin activation through the crosstalk between MTA1/WNT and PI3K/AKT pathways in HTR-8/SVneo cells. (a) HTR-8/SVneo cells were treated with exogenous leptin (0 and 200 ng/ml) for 24 h, and MTA1, WNT1, p-GSK3*β* (Ser9), and p-AKT (Ser473) levels were detected by Western blot. Data are shown as mean ± SD; ^∗^*P* < 0.01 vs. leptin (0 ng/ml). (b) The knockdown efficiencies of MTA1 and WNT1 were analyzed by Western blot analysis and qRT-PCR. ^∗^*P* < 0.05 vs. control. (c) HTR-8/SVneo cells were transfected with MTA1 siRNA or scramble siRNA (Scr) in the presence or absence of 200 ng/ml leptin for 24 h, and Western blot analysis was performed to detect the expression of MTA1, WNT1, p-GSK3*β* (Ser9), and nuclear *β*-catenin. ^∗^*P* < 0.01 vs. control and ^#^*P* < 0.01 vs. leptin. (d) HTR-8/SVneo cells were transfected with WNT1 siRNA or scramble siRNA (Scr) in the presence or absence of 200 ng/ml leptin for 24 h, and Western blot analysis was performed to detect the expression of WNT1, p-GSK3*β* (Ser9), and nuclear *β*-catenin. ^∗^*P* < 0.01 vs. control and ^#^*P* < 0.01 vs. leptin. (e) The knockdown efficiencies of AKT and *β*-catenin were analyzed by Western blot analysis and qRT-PCR. ^∗^*P* < 0.05 vs. control. (f) HTR-8/SVneo cells were transfected with AKT siRNA or scramble siRNA (Scr) in the presence or absence of 200 ng/ml leptin for 24 h, and Western blot analysis was performed to detect the expression of AKT, p-GSK3*β* (Ser9), and nuclear *β*-catenin. ^∗^*P* < 0.01 vs. control and ^#^*P* < 0.01 vs. leptin. All experiments were performed in triplicate.

It is known that *β*-catenin is the pivotal molecule of WNT/*β*-catenin signaling pathway. Thus, we detected whether MTA1/WNT signaling pathway is involved in *β*-catenin activation in HTR-8/SVneo cells. To address this question, we conducted Western blot analysis to detect the expression of MTA1, WNT1, and GSK3*β* in HTR-8/SVneo cells treated with 200 ng/ml leptin for 24 h. As shown in Figure 4(a), leptin exposure significantly improved the protein level of MTA1, WNT1, and p-GSK3*β* (Ser9) when compared with the control. Then, we silenced the expression of MTA1 and WNT1 in HTR-8/SVneo cells using MTA1 siRNA and WNT1 siRNA, respectively. The knockdown efficiencies were confirmed by Western blot analysis and qRT-PCR (Figure 4(b)). Next, we conducted Western blot analysis to examine their downstream effectors. Results showed that MTA1 knockdown inhibited leptin-induced WNT1, p-GSK3*β* (Ser9), and nuclear *β*-catenin expression (Figure 4(c)) while WNT1 knockdown inhibited leptin-induced p-GSK3*β* (Ser9) and nuclear *β*-catenin expression (Figure 4(d)), suggesting that MTA1 locates upstream of WNT1 and WNT1 locates upstream of GSK3*β*. Interestingly, suppressing MTA1 or WNT1 levels did not totally inhibit the level of leptin-induced p-GSK3*β* (Ser9) and nuclear *β*-catenin, suggesting the presence of an MTA1/WNT1-independent mechanism to regulate leptin-induced p-GSK3*β* (Ser9) and *β*-catenin. Recently, there has been reported a “crosstalk” between the WNT/*β*-catenin and PI3K/AKT pathways by GSK3*β*. Considering the role of PI3K/AKT signaling pathway in invasion, we further detected the effect of PI3K/AKT in regulating leptin-induced p-GSK3*β* (Ser9) and activation of *β*-catenin in HTR-8/SVneo cells. As shown in Figure 4(a), leptin exposure increased the protein level of p-AKT (Ser473), while inhibition of AKT by AKT siRNA (Figure 4(e)) reduced leptin-mediated increase in p-GSK3*β* (Ser9) and nuclear *β*-catenin (Figure 4(f)). In a word, our study suggested both MTA1/WNT and PI3K/AKT pathways were involved in the regulation of leptin-mediated activation of *β*-catenin.

### 3.5. MTA1/WNT and PI3K/AKT Pathways Participated in Leptin-Induced HT-R8/SVneo Cell Invasion through Promoting MMP9 Expression

**Figure 5:**
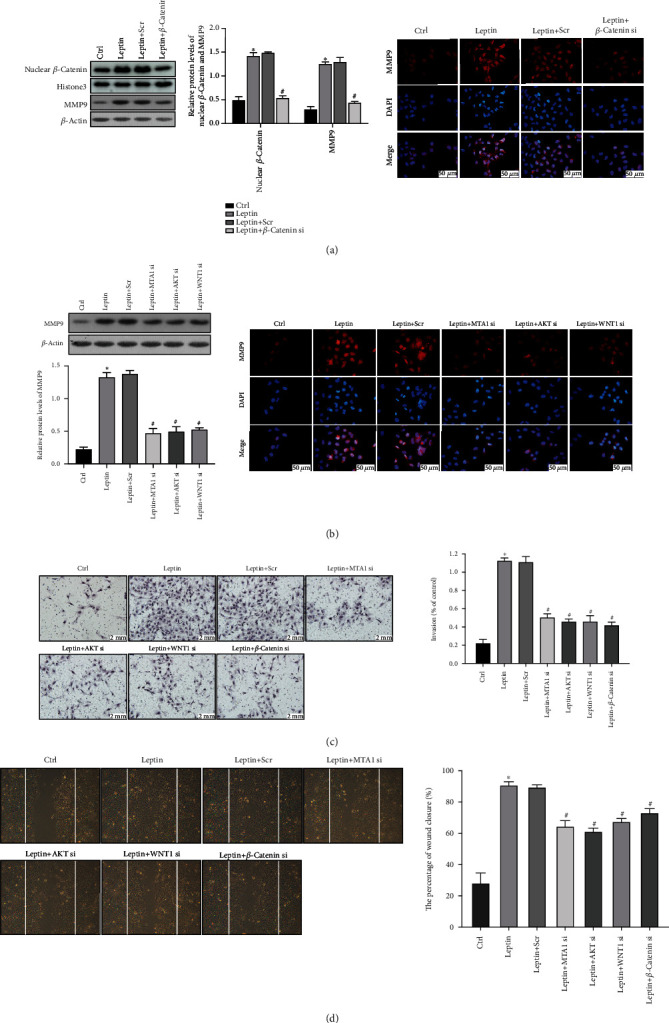
MTA1/WNT and PI3K/AKT pathways participated in leptin-induced HTR-8/SVneo cell invasion through promoting MMP9 expression. (a) Western blot and immunofluorescence staining of MMP9 levels in HTR-8/SVneo cells treated with *β*-catenin siRNA or scramble siRNA (Scr) in the absence or in the presence of 200 ng/ml leptin for 24 h. Scale bar = 50*μ*m. (b) Western blot and immunofluorescence staining of MMP9 levels in HTR-8/SVneo cells treated with MTA1, AKT, and WNT1 in the absence or in the presence of 200 ng/ml leptin for 24 h. Scale bar = 50*μ*m. (c) The results of transwell assay in HTR-8/SVneo cells treated with MTA1 siRNA, AKT siRNA, WNT1 siRNA, *β*-catenin siRNA, or scramble siRNA (Scr) in the absence or in the presence of 200 ng/ml leptin for 24 h. Scale bar = 2mm. Data are shown as mean ± SD; ^∗^*P* < 0.01 vs. Control and ^#^*P* < 0.01 vs. leptin. (d) Results from the wound-healing assay are expressed as the percentage of wound closure. Magnification, 100x. Data are shown as mean ± SD; ^∗^*P* < 0.01 vs. control and ^#^*P* < 0.01 vs. leptin. All experiments were performed in triplicate.

Further, we studied whether MTA1/WNT/GSK3*β*/*β*-catenin and PI3K/AKT/GSK3*β*/*β*-catenin pathways were involved in the expression of MMP9 and the invasion of leptin-induced HTR-8/SVneo cells. Firstly, we constructed *β*-catenin siRNA and verified the knockdown efficiency (Figure 4(e)). By performing Western blot and immunofluorescence staining, we observed that the silence of *β*-catenin reduced the protein levels of MMP9 (Figure 5(a)). In addition, compared with control cells, the expression of MMP9 was also reduced in MTA1 siRNA, AKT siRNA, and WNT1 siRNA cells treated with leptin (Figure 5(b)), indicating that MTA1, AKT, WNT1, and *β*-catenin are involved in the regulation of MMP9. Subsequently, the transwell assay and wound-healing assay were carried out, and we observed that knockdown of AKT, MTA1, WNT1, or *β*-catenin significantly impaired the invasion ability of leptin-induced HTR-8/SVneo cells (Figures 5(c) and 5(d)). Given our results, we conclude that MTA1/WNT/GSK3*β*/*β*-catenin and PI3K/AKT/GSK3*β*/*β*-catenin pathways promote the expression of MMP9 and play an indispensable role in leptin-induced cell invasion.

## 4. Discussion

Studies have showed that the establishment and maintenance of biological pregnancy required moderate invasion of trophoblast cells into the endometrial. Leptin, which is elevated in pregnancy, is indispensable in the procession of trophoblast invasiveness [16, 34], and leptin-R was detected to be strongly expressed in the distal extravillous cytotrophoblastic cells of cell columns [35]. Consistent with previous studies, the transwell assay showed the close connection between the leptin and trophoblast invasiveness and further revealed that leptin can promote the invasion of HTR-8/SVneo cells in a dose- and time-dependent manner.

The process of trophoblastic cell invasion involves matrix metalloproteinases (MMPs) to mediate the degradation of extracellular matrix (ECM) [36, 37]. A previous study reported that inhibited expression of MMP9 can decrease the invasive capability of trophoblasts [38]. Additionally, at the embryo implantation site by human and mouse trophoblasts, the high expression of MMP9 has also been indicated to be involved in the invasive behavior [39]. On the contrary, the deficiency of MMP9 in mouse embryos brings about failure in trophoblast differentiation and invasion shortly after implantation [39], implying that MMP9 is an important factor in trophoblast invasion. As it is mentioned above, we observed that leptin stimulation can promote the expression of MMP9 in a concentration/time-dependent manner and identify MMP9 as the modulator to the proinvasion effect of leptin on HTR-8/SVneo cells.

Increasing evidences have shown that *β*-catenin could enhance the invasion of trophoblast, while inhibited *β*-catenin could damage the function of trophoblast [40, 41]. In the present study, Western blot analysis and immunofluorescence staining revealed that the nuclear protein expression of *β*-catenin in leptin-induced HTR-8/SVneo cells was statistically higher than the control group, proving that leptin may induce the invasion of HTR-8/SVneo cells via promoting the nuclear translocation of *β*-catenin.

Besides, researches demonstrated that the level of *β*-catenin is the core molecule to the activation of WNT/*β*-catenin signaling pathway [28]. With the absence of WNT, cytoplasmic *β*-catenin is phosphorylated by a multiprotein destruction complex which is composed of glycogen synthase kinase 3*β* (GSK3*β*), casein kinase 1 (CK1), tumor suppressor adenomatous polyposis coli (APC) gene product, and scaffolding protein Axin, leading to ubiquitination and subsequent degradation by the proteasomal system [42]. Instead, when in the presence of a WNT ligand, binding of WNT to the Frizzled receptor inhibits the multiprotein destruction complex and then prevents it from phosphorylating *β*-catenin. Thus, *β*-catenin accumulates in the cytoplasm and travels to the nucleus to regulate transcription of target genes [43]. A recent study has demonstrated that MTA1 is expressed in human trophoblast cells at a rather high level [32], revealing that MTA1 has a potential role in normal human trophoblast cell. In this study, we observed a significant increase of MTA1, WNT1, and p-GSK3*β* (Ser9) in leptin-induced HTR-8/SVneo cells and found a significant repression of WNT1, p-GSK3*β* (Ser9), and nuclear *β*-catenin when knocking down MTA1 by siRNA, suggesting that MTA1 may mediate WNT/*β*-catenin signaling in HTR-8/SVneo cells. Intriguingly, silencing MTA1 or WNT1 did not totally inhibit the expression of p-GSK3*β* (Ser9) and nuclear *β*-catenin in leptin-treated HTR-8/SVneo cells. Thus, we speculate that there may be other MTA1/WNT1-independent mechanisms to regulate leptin-induced p-GSK3*β* (Ser9) and nuclear *β*-catenin.

PI3K/AKT pathway, another important pathway to mediate invasion [44–46], has been described to be activated by leptin in the placenta [47, 48]. Generally, activated AKT, which is phosphorylated at the Ser473 site, induces the inactivation of GSK3*β* through phosphorylating GSK-3*β* at Ser9, thereby inhibiting the degradation of *β*-catenin, leading to the accumulation of *β*-catenin in the cytoplasm [49, 50]. In our present study, we demonstrated the crosstalk between the WNT/*β*-catenin and PI3K/AKT pathway via GSK3*β* in HTR-8/SVneo cells. By performing Western blot, we observed a significant increase of p-AKT (Ser473) in leptin-induced HTR-8/SVneo cells, and inhibition of AKT reduced the expression level of p-GSK3*β* (Ser9) and nuclear *β*-catenin. Besides, we revealed that MTA1/WNT/GSK3*β*/*β*-catenin and PI3K/AKT/GSK3*β*/*β*-catenin pathways promoted the expression of MMP9 and augmented leptin-induced cell invasion by conducting loss-of-function analysis, enriching the exploration of cell invasion.

## 5. Conclusions

**Figure 6:**
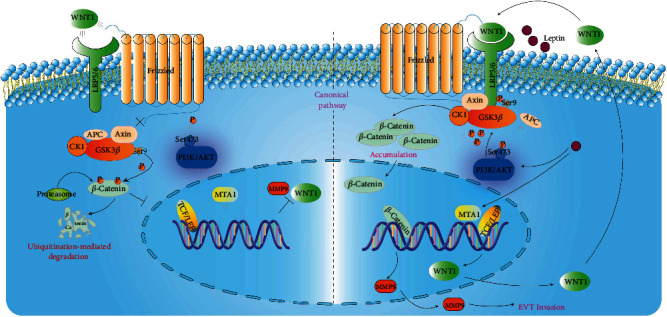
Schematic model of the role of leptin in HTR-8/SVneo cell invasion.

In summary, our study discovered a link between the leptin and MMP9 in leptin-induced HTR-8/SVneo cell invasion and illuminated a potential mechanism that leptin promoted MMP9 upregulation via the crosstalk between MTA1/WNT and PI3K/AKT pathways (Figure 6), which will provide a new therapeutic target for the clinical prevention and treatment of trophoblast invasion in the future.

## Figures and Tables

**Table 1:** The sequences of the primers used in qRT-PCR.

Name	Sequence
MTA1	F 5′-TGGAGAACCCGGAAATGGTG-3′
	R 5′-TCCAGGTAGGACTTGAGCGA-3′

WNT1	F 5′-CGATGGTGGGGTATTGTGAA-3′
	R 5′-GGAACTGCCACTTGCACTC-3′

AKT1	F 5′-GGACAAGGACGGGCACATTA-3′
	R 5′-CGACCGCACATCATCTCGTA-3′

*β*-Catenin	F 5′-GCAGCGACTAAGCAGGAAGG-3′
	R 5′-CTGTCACCAGCACGAAGGAC-3′

MMP9	F 5′-TTCAGGGAGACGCCCATTTC-3′
	R 5′-AACCGAGTTGGAACCACGAC-3′

*β*-Actin	F 5′-GAAGAGCTACGAGCTGCCTGA-3'
	R 5′-CAGACAGCACTGTGTTGGCG-3′

## Data Availability

All data generated or analyzed during this study are included in this article.
